# The development and validation of a decision aid to enhance shared decision‐making for the management of actinic keratosis

**DOI:** 10.1002/ski2.388

**Published:** 2024-04-23

**Authors:** Geoffrey Brent, Caroline Beardmore, Kate Mayers, Alberto Barea, Venura Samarasinghe, Vanessa Pinder, Julia Soo, Victoria Akhras, Zainab Jiyad

**Affiliations:** ^1^ Department of Dermatology Epsom and St Helier University Hospitals NHS Trust Carshalton Surrey UK; ^2^ St George's University of London Medical School London UK; ^3^ Department of Dermatology Kingston Hospitals NHS Foundation Trust Kingston Upon Thames UK; ^4^ Department of Dermatology St George's University Hospitals NHS Trust Tooting UK; ^5^ Population Health Research Institute St George's University of London London UK

## Abstract

Actinic keratoses (AKs) are common pre‐malignant lesions. There are numerous management options including active surveillance, multiple topical therapies, cryotherapy, curettage and cautery, and photodynamic therapy, each with their own risks, benefits and efficacy. Best practice currently involves shared decision‐making between patient and clinician, particularly in the setting of multiple management options. Patient decision aids have been shown to be beneficial in the shared decision‐making process. In view of this, we have developed and validated a decision aid for the management of AKs, in concordance with the International Patient Decision Aids Standards.

## Graphical abstract

Actinic keratoses (AKs) are common pre‐malignant lesions for which there are numerous management options, each with their own risks, benefits and efficacy. Best practice currently involves shared decision‐making between patient and clinician, particularly in the setting of multiple management options. Patient decision aids have been shown to be beneficial in the shared decision‐making process. In view of this, we have developed and validated a novel decision aid for the management of AKs.

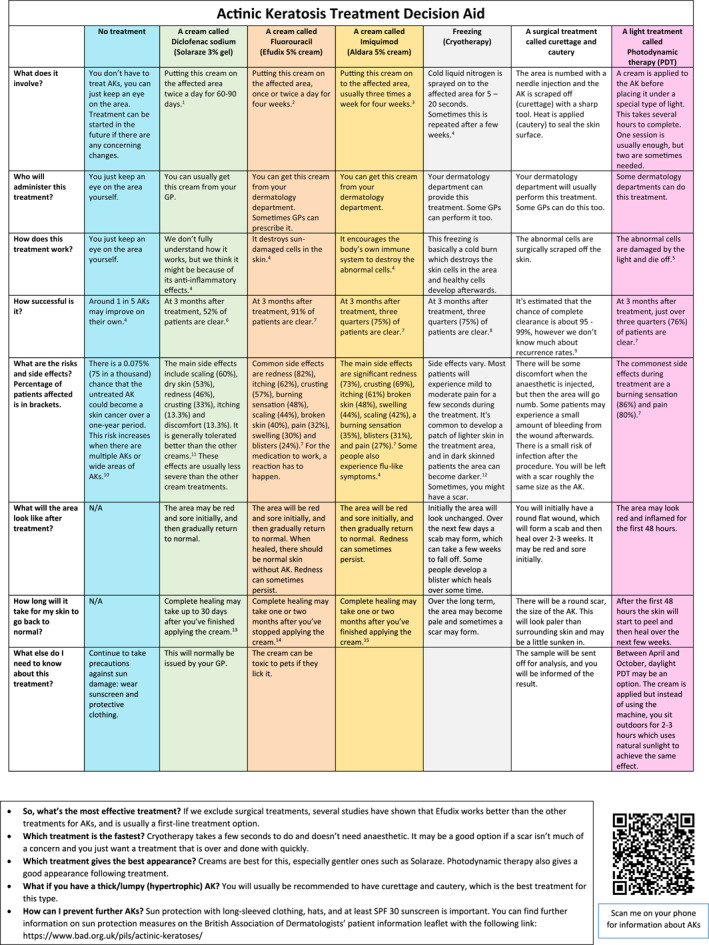

## 1 INTRODUCTION

Actinic keratoses (AKs) are pre‐malignant keratotic lesions typically found on sun‐exposed areas of skin. They are highly prevalent, with nearly a quarter of adults aged over 60 having at least one AK.^1^ Management options include active surveillance, topical therapies such as diclofenac sodium, 5‐fluorouracil and imiquimod, focal destructive treatment such as cryotherapy or curettage and cautery, and photodynamic therapy.^1^, ^2^ 5‐fluorouracil and cryotherapy are commonly used first‐line. A number of studies have evaluated these management options. Jansen et al conducted a randomised controlled trial comparing 5% fluorouracil cream, 5% imiquimod cream, methyl aminolevulinate photodynamic therapy (MAL‐PDT), and 0.015% ingenol mebutate gel.^2^ The primary outcome was the proportion of patients with a reduction of 75% or more in the number of actinic keratosis lesions from baseline to 12 months post‐treatment, with fluorouracil found to be the most effective. Szeimies et al compared MAL‐PDT with cryotherapy in a prospective, randomised study, finding that MAL‐PDT had a similar response rate to that of cryotherapy, but superior cosmetic results and high patient satisfaction.^3^ The British Association of Dermatologists (BAD) published guidelines for the management of AKs in 2017.^1^ In addition to these treatments, active surveillance is also credited as a valid management option for AKs in lower risk groups. Costing information for the topical treatments are as follows: diclofenac sodium = £38.30 per 50 g tube, 5‐fluorouracil = £32.90 per 40 g tube, and imiquimod 5% = £48.60 per 12 sachets.^4^ For the procedures, cryotherapy = £142.00 per patient, curettage and cautery = £142.00 per treatment, and PDT = £458 per treatment as per the 2023—2025 National Health Service (NHS) Payment Scheme, excluding the market forces factor.^5^

The risks and benefits for each treatment option vary widely, and tailoring the management to the individual patient is key to optimising outcomes and patient satisfaction. Best practice involves shared decision making between patient and clinician, particularly in this setting where there are multiple management options. Patient decision aids (PDA) have been shown to be beneficial in the shared decision‐making process, enabling patients to consider the advantages and disadvantages of the available treatment options whilst developing their own knowledge.^6^, ^7^, ^8^

## 2 REPORT

A clinical need for a decision aid on AK management for patients was identified. Between December 2022—August 2023 we developed a novel PDA amongst clinicians and dermatology nurses across the South West London region, in concordance with the International Patient Decision Aids Standards.^9^, ^10^ Ethics approval was not required. A literature search was undertaken to ensure evidence‐based information was provided on the PDA.

Following creation of the initial draft PDA, alpha testing was conducted with feedback from clinicians and patients. A questionnaire was provided to five consecutive patients to establish whether the PDA was easy to read and understand, contained an appropriate volume of information, and inviting any further comments (Supplementary Figure S1). This prompted minor formatting changes including creating a colour version to improve readability, and removing a paragraph explaining what AKs are and replacing this with a quick‐response (QR) code link to the BAD information leaflet on AKs. As the average reading age of the UK has been estimated at 11–16 years old, we used simple language and utilised the Gunning Fog Index to confirm readability of the text for someone aged 12.^11^

**FIGURE 1:**
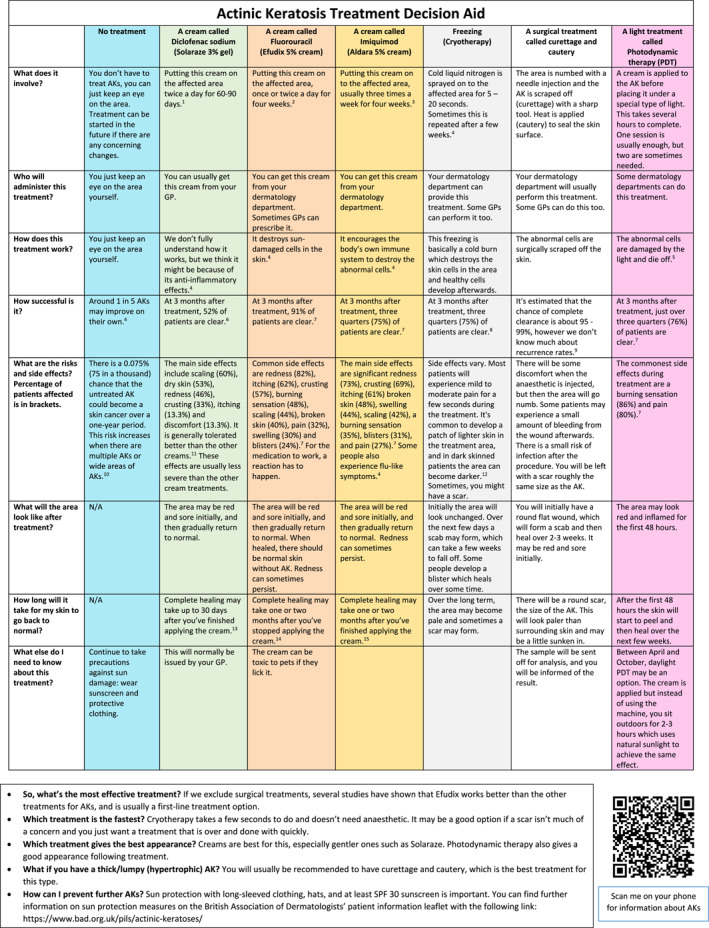
Actinic keratosis patient decision aid.

The final PDA (Figure 1) underwent beta testing using two validated outcome measures, the Decisional Conflict Scale (DCS) and the nine‐item Shared Decision‐Making Questionnaire (SDM‐Q‐9).^12^, ^13^ We used the PDA in 21 consecutive patients across three separate Hospital Trusts in dermatology clinics for patients over 18 years of age diagnosed with one or more AKs clinically or histologically. Patients where there was any suspicion of squamous cell carcinoma, or had tender or hypertrophic AKs were excluded. The DCS questionnaire was first given to the patients following diagnosis, but before the discussion of management took place. The patient was given time to read the PDA outside the clinic room. After the consultation was completed and a management plan agreed upon, a post‐consultation DCS questionnaire was performed. The SDM‐Q‐9 was completed last to establish the effect the PDA had on shared decision‐making.

Data were obtained from 21 consecutive patients who met the inclusion criteria. One patient was subsequently excluded due to filling in the questionnaires incorrectly. 10/20 (50%) of patients were men. 15 patients had data on their age, with mean ages 69 years for men and 59 years for women. The majority of patients (75%) indicated a high level of decisional conflict prior to using the PDA, with a total mean DCS score of 52.97 (95% CI 43.2–62.8), where an increasing value corresponds with increasing decisional conflict. Following use of the PDA, the total mean DCS score improved to 12.97 (95% CI 8.0–17.9). This represents a significant reduction in DCS score (*p* < 0.0001), with the majority of responses as “strongly agree” and “agree” that the PDA facilitated their management decision. The largest improvement between pre‐ and post‐PDA scores was seen in the “uncertainty” subscale, demonstrating the PDA improves the clarity, certainty and ease in which patients are able to make a decision on their treatment. The total SDM‐Q‐9 score was 791/900 (87.9%) which indicates a high level of shared decision‐making following use of the PDA. No further changes were made to the PDA. References used within the PDA are included separately (Supplementary Table S1).

There are some limitations to our study. The literature on the management of AKs is heterogenous with varying inclusion and exclusion criteria, endpoints, and outcomes measures. We included only high‐quality prospective studies and randomised controlled trials, and used our judgement in assessing these when creating the PDA. We excluded topical tirbanibulin (Klisyri®) due to limited experience among our clinicians and lack of long‐term data, and 5‐fluorouracil plus salicylic acid (Actikerall®) due to its uncommon use. The PDA was validated in the secondary care setting, and further studies would be required to assess its use in primary care. However, we suggest it may be useful at the interface between the two such as with Advice and Guidance services. Finally, this patient decision aid is not intended to be used for all patients, and should be used when appropriate for the clinical context.

Actinic keratoses can be managed in various ways and the information given to patients can be overwhelming which may make the decision more challenging. A PDA given to the patient can reduce decisional conflict and improve the shared decision‐making process. We suggest that this novel validated decision aid can be used in various settings to facilitate informed care, particularly in secondary care in dermatology specialist nurse and physician‐led clinics. Additionally, the PDA can be used at the interface of primary and secondary care when responding to Advice and Guidance queries. Further studies would be useful to assess the PDA in a primary care setting, and on its effectiveness in Advice and Guidance services.

## CONFLICT OF INTEREST STATEMENT

None to declare.

## AUTHOR CONTRIBUTIONS

**Geoffrey Brent**: Conceptualization (supporting); data curation (equal); formal analysis (equal); investigation (equal); methodology (equal); project administration (lead); validation (equal); writing—original draft (lead); writing—review and editing (equal). **Caroline Beardmore**: Project administration (supporting); writing—original draft (equal); writing—review and editing (supporting). **Kate Mayers**: Data curation (equal); investigation (supporting); writing—review and editing (equal). **Alberto Barea**: Conceptualization (supporting); methodology (equal); writing—review and editing (equal). **Venura Samarasinghe**: Conceptualization (supporting); methodology (equal); writing—review and editing (equal). **Vanessa Pinder**: Conceptualization (supporting); methodology (supporting); writing—review and editing (equal). **Julia Soo**: Conceptualization (supporting); methodology (supporting); writing—review and editing (equal). **Victoria Akhras**: Conceptualization (equal); data curation (equal); investigation (equal); methodology (equal); validation (supporting); writing—original draft (supporting); writing—review and editing (equal). **Zainab Jiyad**: Conceptualization (lead); formal analysis (equal); investigation (supporting); methodology (equal); project administration (supporting); supervision (lead); validation (equal); writing—review and editing (equal).

## FUNDING INFORMATION

This article received no specific grant from any funding agency in the public, commercial, or not‐for‐profit sectors.

## ETHICS STATEMENT

Not applicable.

## Supporting information

Figure S1

Table S2

## Data Availability

The data that support the findings of this study are available from the corresponding author upon reasonable request.
