# Correction to: The Development and Validation of a Decision Aid to Enhance Shared Decision-Making for the Management of Actinic Keratosis

**DOI:** 10.1093/skinhd/vzaf003

**Published:** 2025-02-11

This is a correction to: Geoffrey Brent, Caroline Beardmore, Kate Mayers, Alberto Barea, Venura Samarasinghe, Vanessa Pinder, Julia Soo, Victoria Akhras, Zainab Jiyad, The Development and Validation of a Decision Aid to Enhance Shared Decision-Making for the Management of Actinic Keratosis, *Skin Health and Disease*, Volume 4, Issue 3, June 2024, https://doi.org/10.1002/ski2.388

In Figure 1, the cell in row ‘What are the risks and side effects’ and column ‘No treatment’, which reads: “There is a 0.075% (75 in a thousand chance)” should read instead: “There is a less than 1% chance”.

The emendation has been made in the article.

