# Supplementary material for: The development and validation of a decision aid to enhance shared decision‐making for the management of actinic keratosis
Source: Skin Health Dis. 2024 Apr 23;4(3):e388. doi: 10.1002/ski2.388 (PMC11150750; doi:10.1002/ski2.388)
Supplement: Supplementary file 1 — Figure S1 [file SKI2-4-e388-s001.pdf]

Supplementary Figure 1: Alpha testing patient questionnaire

Actinic Keratosis Patient Decision Aid Questionnaire

1. Is the **purpose** of the Patient Decision Aid clear? Yes No

2. Would it be useful to read the Patient Decision Aid leaflet in your own time *before* deciding on treatment for your actinic keratosis with your clinician? Yes No

3. Do you find the Patient Decision Aid easy to **read**? Yes No

   If **no**, what could we do to make it easier to read?

   ....

   ....

   ....

   ....

4. Do you find the Patient Decision aid easy to **understand**? Yes No

   If **no**, please let us know what it was that you didn't understand and how you think we could improve it.

   ....

   ....

   ....

   ....

5. Do you think there is (please tick one option):
   a. Too little information ☐
   b. Just the right amount of information ☐
   c. Too much information ☐

6. Do you have any other comments or suggestions?

   ....

   ....

   ....

   ....

Thank you, we really appreciate your feedback.
