# Supplementary material for: The development and validation of a decision aid to enhance shared decision‐making for the management of actinic keratosis
Source: Skin Health Dis. 2024 Apr 23;4(3):e388. doi: 10.1002/ski2.388 (PMC11150750; doi:10.1002/ski2.388)
Supplement: Supplementary file 2 — Table S2 [file SKI2-4-e388-s002.docx]

| **References for Patient Decision Aid** | |
| --- | --- |
| **1** | Diclofenac Sodium. In: Joint Formulary Committee. British National Formulary [Internet]. London: British Medical Association and Royal Pharmaceutical Society of Great Britain; [updated 2022 Oct 16; cited 2022 Dec 03]. Available from: https://bnf.nice.org.uk/drugs/diclofenac-sodium/ |
| **2** | Fluorouracil. In: Joint Formulary Committee. British National Formulary [Internet]. London: British Medical Association and Royal Pharmaceutical Society of Great Britain; [updated 2022 Oct 16; cited 2022 Dec 03]. Available from: https://bnf.nice.org.uk/drugs/fluorouracil/ |
| **3** | Imiquimod. In: Joint Formulary Committee. British National Formulary [Internet]. London: British Medical Association and Royal Pharmaceutical Society of Great Britain; [updated 2022 Oct 16; cited 2022 Dec 03]. Available from: https://bnf.nice.org.uk/drugs/imiquimod/ |
| **4** | De Berker D, McGregor JM, Mohd Mustapa MF, Exton LS, Hughes BR, McHenry PM, Gibbon K, Buckley DA, Nasr I, Duarte Williamson CE, Swale VJ. British Association of Dermatologists’ guidelines for the care of patients with actinic keratosis 2017. British Journal of Dermatology. 2017 Jan;176(1):20-43. |
| **5** | Dirschka T, Radny P, Dominicus R, Mensing H, Brüning H, Jenne L, Karl L, Sebastian M, Oster‐Schmidt C, Klövekorn W, Reinhold U. Photodynamic therapy with BF‐200 ALA for the treatment of actinic keratosis: results of a multicentre, randomized, observer‐blind phase III study in comparison with a registered methyl‐5‐aminolaevulinate cream and placebo. British Journal of Dermatology. 2012 Jan;166(1):137-46. |
| **6** | Zane C, Facchinetti E, Rossi MT, Specchia C, Calzavara‐Pinton PG. A randomized clinical trial of photodynamic therapy with methyl aminolaevulinate vs. diclofenac 3% plus hyaluronic acid gel for the treatment of multiple actinic keratoses of the face and scalp. British Journal of Dermatology. 2014 May;170(5):1143-50. |
| **7** | Jansen MH, Kessels JP, Nelemans PJ, Kouloubis N, Arits AH, van Pelt HP, Quaedvlieg PJ, Essers BA, Steijlen PM, Kelleners-Smeets NW, Mosterd K. Randomized trial of four treatment approaches for actinic keratosis. New England Journal of Medicine. 2019 Mar 7;380(10):935-46. |
| **8** | Szeimies RM, Karrer S, Radakovic-Fijan S et al. Photodynamic therapy using topical methyl 5-aminolevulinate compared with cryotherapy for actinic keratosis: a prospective, randomized study. J Am Acad Dermatol 2002; 47:258–62. |
| **9** | Uhlenhake EE. Optimal treatment of actinic keratoses. Clin Interv Aging. 2013;8:29-35. |
| **10** | Werner RN, Sammain A, Erdmann R, Hartmann V, Stockfleth E, Nast A. The natural history of actinic keratosis: a systematic review. British Journal of Dermatology. 2013 Sep;169(3):502-18. |
| **11** | Segatto MM, Dornelles SI, Silveira VB, Frantz Gde O. Comparative study of actinic keratosis treatment with 3% diclofenac sodium and 5% 5-fluorouracil. An Bras Dermatol. 2013 Sep-Oct;88(5):732-8. |
| **12** | Arisi M, Guasco Pisani E, Calzavara-Pinton P, Zane C. Cryotherapy for Actinic Keratosis: Basic Principles and Literature Review. Clin Cosmet Investig Dermatol. 2022 Mar 5;15:357-365. |
| **13** | Almirall Limited. Solaraze 3% gel SmPC. 2021 [Cited 2022 Dec 3]. Available from: https://www.medicines.org.uk/emc/product/6385/smpc |
| **14** | Mylan. Efudix 5% cream SmPC. 2022 [Cited 2022 Dec 3]. Available from: https://www.medicines.org.uk/emc/product/9260/smpc |
| **15** | Meda Pharmaceuticals. Aldara 5% cream SmPC. 2017 [Cited 2022 Dec 3]. Available from: https://www.medicines.org.uk/emc/product/823/smpc |
